# Metabolomics biomarkers for precision psychiatry

**DOI:** 10.3389/fpsyt.2026.1736206

**Published:** 2026-02-13

**Authors:** Daniele Cavaleri, Carlo Bassetti, Giorgio Cucchi, Pasquale De Fazio, Renato de Filippis, Umberto Albert, Luca Pellegrini, Giuseppe Carrà, Francesco Bartoli

**Affiliations:** 1 School of Medicine and Surgery, University of Milano-Bicocca, Monza, Italy; 2 Department of Health Sciences, University “Magna Graecia” of Catanzaro, Catanzaro, Italy; 3 Department of Medicine, Surgery and Health Sciences, University of Trieste, Trieste, Italy

**Keywords:** anxiety disorders, biomarkers, bipolar disorder, major depressive disorder, metabolomics, obsessive-compulsive disorder, post-traumatic stress disorder, schizophrenia

## Abstract

Mental disorders remain diagnosed primarily through symptom-based classification systems that overlook biological heterogeneity, preventing the identification of mechanistically distinct patient subgroups and precluding pathophysiology-guided treatment selection. Metabolomics offers a promising pathway towards precision psychiatry by capturing dynamic biochemical readouts at the functional endpoint of the omics cascade, integrating genetic, environmental, and pharmacological influences on cellular metabolism. Over the past 15 years, untargeted and targeted metabolomics studies using nuclear magnetic resonance spectroscopy and mass spectrometry have identified consistent patterns of metabolic dysregulation across psychiatric disorders, particularly involving amino acid metabolism, lipid signaling, energy homeostasis, and oxidative stress pathways. Schizophrenia presents disruptions in arginine and proline metabolism, glutathione metabolism, and energy-related processes. Bipolar disorder shows perturbations in branched-chain and aromatic amino acids, kynurenine pathway, and tricarboxylic acid cycle dysfunction with phase-specific metabolic signatures. Major depressive disorder exhibits widespread alterations in amino acid turnover, bioenergetic processes, membrane lipid homeostasis, and glutamate-GABA cycling, with treatment-responsive metabolic changes. Despite these advances, substantial challenges remain: heterogeneous findings with disorder overlap, limited replication cohorts, predominance of cross-sectional designs, confounding by medication and lifestyle factors, pre-analytical variability, and high-dimensional data complexity. Future research requires harmonized multi-site protocols, longitudinal validation studies, multi-platform analytical approaches, integration with genomics, proteomics, and digital phenotyping, and implementation of artificial intelligence frameworks to enhance phenotype discrimination and predictive accuracy. In this mini-review, we provide an overview of current methodologies, major findings, strengths, challenges, and emerging directions in psychiatric metabolomics, with the goal of facilitating the translation of metabolomic insights into clinically applicable, personalized psychiatric treatment.

## 1 Background

Mental disorders encompass a wide spectrum of heterogenous conditions whose etiologies involve biological, environmental, and psychosocial factors (1). Despite major advances in neuroscience, clinical psychiatry keeps relying on symptom-based diagnostic systems that categorize patients by clusters of observable behaviors and self-reported symptoms but overlook biological heterogeneity, contributing to diagnostic uncertainty and unsatisfactory clinical management (2–4).

The concept of precision psychiatry aims to move beyond the “one-size-fits-all” approach by integrating multidimensional data – including omics, neuroimaging, and digital phenotyping – to stratify patients into biologically meaningful subgroups, predict treatment response, and monitor disease trajectories (5). In this perspective, metabolomics holds particular promise. Metabolites indeed represent the end products of gene-environment interactions and reflect real-time biochemical activity across interconnected pathways (6). Metabolomics thus sits at the downstream end of the omics cascade and provides a sensitive functional readout of cellular and systemic metabolism and its changes in response to physiological and pathological states, environmental exposures, and pharmacological interventions (7, 8). This offers a dynamic readout ideal for stratification and monitoring. Alterations in amino acid metabolism, lipid signaling, energy homeostasis, and oxidative stress markers have been consistently reported in schizophrenia (SCZ) and affective disorders, suggesting that metabolomics features can reveal disease-relevant pathway perturbations (9, 10).

Early metabolomics investigations in psychiatric research were largely exploratory, focusing on identifying candidate metabolites in blood, cerebrospinal fluid (CSF), and urine from small cohorts (11–13). While these studies established proof-of-concept for disease-associated metabolic dysregulation, they often lacked replication, employed heterogeneous analytical methods, and were limited by insufficient sample sizes, hindering clinical translation. More recent efforts have emphasized standardized protocols, targeted validation of previously reported biomarkers, and improved reproducibility. Nonetheless, several challenges remain: pre-analytical and analytical variability, the lack of large-scale longitudinal studies, and the scarce integration of metabolomics data with other omics layers represent key issues.

In this mini-review, we synthesize main findings in psychiatric metabolomics, also providing a critical methodological overview and appraising the field’s major strengths, barriers and priorities for future research. Our overarching goal is to propose a clinically oriented framework that supports progression from biomarker discovery to validation and eventual clinical translation, accelerating the development of metabolomics-informed, personalized psychiatric care.

## 2 Metabolomics methodology: a brief overview

Metabolomics is defined as the systematic analysis of low-molecular-weight biological compounds (<1.5 kDa) within a specific biological system (6). Thus, the metabolome encompasses the complete set of metabolites – including metabolic intermediates, hormones, signaling molecules, and secondary metabolites – found within a cell, a tissue, a biofluid, or the whole organism (6). These compounds include different chemical classes such as amino acids, lipids, sugars, nucleotides, and organic acids. Unlike the relatively static genome and transcriptome, the metabolome is highly dynamic and time-dependent, changing from second to second in response to gene expression, physiological states, environmental perturbations, and pathophysiological stimuli (14, 15). This dynamic nature puts metabolomics at the functional endpoint of the omics cascade, providing a direct readout of cellular activity and phenotypic expression that integrates upstream genomics, transcriptomics, and proteomics information (16).

A critical aspect of metabolomics research is the choice of the specimen, which varies according to disease and practicality. Blood (whether plasma or serum) and urine represent the most commonly used biofluids in metabolomics due to their non-invasive collection and rich metabolite content reflecting systemic metabolic changes that may be associated with psychiatric conditions. Nonetheless, peripheral metabolites are subject to influence by systemic processes and diet, making them imperfect proxies for brain biochemistry (17). On the other hand, CSF, by virtue of its proximity to central nervous system processes, has been particularly valuable (13), but its collection is invasive and costly, hence less feasible for large cohorts. Saliva represents an emerging biofluid offering completely non-invasive sampling (18), while dried blood spots enable simplified collection, storage, and transport while maintaining metabolite stability (19). The pre-analytical and analytical handling of samples – including fasting state, collection timing, storage conditions, and batch processing – must be rigorously controlled and standardized, as they can significantly affect metabolite profiles (20).

Metabolomics studies employ analytical platforms that detect and quantify these small molecules. The two primary analytical platforms in metabolomics are nuclear magnetic resonance (NMR) spectroscopy and mass spectrometry (MS). Each platform offers complementary strengths and limitations in sensitivity, quantification, and metabolite coverage. NMR detects the most abundant metabolites (typically ≥1 μM) (21) and provides excellent technical reproducibility (22). However, NMR suffers from limited sensitivity and spectral resolution, often resulting in peak overlap that complicates metabolite identification (23). In contrast, MS platforms – including liquid chromatography-MS (LC-MS) and gas chromatography-MS (GC-MS) – detect metabolites that are readily ionizable and offer superior sensitivity, resolution (10³ to 10^4^), and dynamic range compared to NMR (24). Nonetheless, MS platforms are susceptible to technical variability (25). Since many metabolites are uniquely identified by either NMR or MS alone, the combination of NMR and MS in multi-platform approaches significantly improves metabolome coverage (26).

Metabolomics approaches can be broadly divided into two complementary strategies that differ in scope, methodology, and application, i.e., untargeted and targeted analyses (27, 28). Untargeted metabolomics aims to achieve a comprehensive overview of the metabolome by detecting as many metabolites as possible within a biological sample, without prior assumptions about their identity (6). Hence, untargeted metabolomics is suited for hypothesis generation, biomarker discovery, and characterization of global metabolic perturbations (29, 30). However, it faces limitations related to metabolite identification, high-dimensional outputs, and quantitative accuracy, which may hinder reproducibility across studies (31). On the other hand, targeted metabolomics focuses on the selective and quantitative analysis of predefined sets of metabolites (6). Targeted metabolomics offers high sensitivity, specificity, and reproducibility, making it particularly suitable for hypothesis-driven studies and clinical or translational applications (32, 33). Targeted analysis also represents an important part of a metabolomics workflow to validate and expand upon results from untargeted analysis. The main drawback of targeted metabolomics is that only known metabolites with available reference standards can be reliably measured (34). An overview of key methodological aspects in metabolomics research is reported in **Table 1**.

**Table 1:** Overview of metabolomics research methodology.

Feature	Options	Strengths	Limitations
Sample type	Blood (plasma/serum)	Easily accessible Rich metabolite content Systemic overview	Indirect proxy for brain metabolism Influenced by systemic processes, environmental factors, and diet
	Urine	Non-invasive Reflects systemic metabolic turnover	Strongly affected by diet, hydration, and circadian rhythm
	Cerebrospinal fluid (CSF)	Closest to central nervous system processes Valuable for psychiatric research	Invasive Costly Limited feasibility in large cohorts
	Saliva	Non-invasive Emerging biomarker source	Lower metabolite concentrations Less validated
	Dried blood spots	Simple collection, storage, and transport Stability of metabolite content	Reduced metabolite coverage compared to plasma/serum
Analytical platforms	Nuclear magnetic resonance (NMR) spectroscopy	Quantitative Good reproducibility	Limited sensitivity; spectral overlap Detects abundant metabolites (≥1 μM)
	Mass spectrometry (MS)	High sensitivity High resolution Broad coverage	Technical variability Higher per-sample fees and more complex preparation than NMR
Approaches	Untargeted metabolomics	Broad coverage Hypothesis-generating	Challenges in identification High-dimensional data Lower reproducibility
	Targeted metabolomics	Sensitive, specific, reproducible Validates untargeted findings Usually less expensive than untargeted metabolomics	Restricted to known metabolites Need for reference standards

## 3 Findings from metabolomics studies across main mental disorders: state-of-the-art

Over the last 15 years, an increasing number of both untargeted and targeted metabolomics investigations have been conducted, identifying recurring metabolite alterations implicated in the pathophysiology of mental disorders (**Table 2**) (7, 9, 35–38). These alterations can often be clustered into some definite biological categories, suggesting that, rather than representing random scattered changes, they converge within specific functional networks to indicate systematic disruptions across key cellular systems.

**Table 2:** Main classes of metabolites and individual molecules relevant to psychiatric disorders, with their putative role.

Metabolite class	Main metabolites	Relevance to psychiatry
Amino acids and derivatives	Tryptophan, tyrosine, glutamate, GABA, glycine, serine	Neurotransmitter precursors and regulators; key roles in depression, schizophrenia, anxiety, autism
Neurotransmitters and neuromodulators	Dopamine, serotonin, norepinephrine, acetylcholine, histamine	Central to mood regulation, cognition, and psychosis
Energy metabolism intermediates	Glucose, lactate, pyruvate, TCA intermediates (e.g. citrate, α-ketoglutarate)	Reflect mitochondrial and metabolic function; altered in depression, bipolar disorder, schizophrenia
Lipid metabolites	Phospholipids, sphingolipids, fatty acids, endocannabinoids	Affect membrane integrity, signaling, and inflammation; relevant in mood and neurodegenerative disorders
Oxidative stress and redox markers	Glutathione, cysteine, methionine, 8-OHdG, NAD^+^/NADH ratio	Indicators of cellular stress and redox imbalance seen in psychiatric conditions
Tryptophan catabolites	Kynurenine, kynurenic acid, quinolinic acid	Modulate NMDAR activity; implicated in depression, schizophrenia, and neuroinflammation
Hormones and steroid metabolites	Cortisol, pregnenolone, testosterone, estrogens	Reflect neuroendocrine regulation and stress response; key in depression and anxiety
Inflammation-related metabolites	Cytokine-induced metabolic markers (e.g., indole derivatives, nitric oxide-related compounds)	Immune-brain interaction markers in psychoneuroimmunology
Gut microbiota-derived metabolites	Short-chain fatty acids (butyrate, propionate), indoles, TMAO	Link gut-brain axis to mood and cognition
Nucleotide and purine metabolites	Adenosine, uric acid, ATP/ADP ratios	Energy and signaling balance, often dysregulated in mood and stress disorders

8-OHdG, 8-hydroxydeoxyguanosine; GABA, γ-aminobutyric acid; NAD^+^, Nicotinamide adenine dinucleotide – oxidized form; NADH, Nicotinamide adenine dinucleotide – reduced form; NMDAR, N-methyl-D-aspartate (NMDA) receptor; TCA, tricarboxylic acid; TMAO, trimethylamine-N-oxide.

In SCZ, metabolomics has revealed consistent patterns of dysregulation involving neurotransmission, oxidation, membrane integrity, and bioenergetics (39–43). Pathway enrichment analyses reported that the most prominently affected processes in SCZ are those related to amino acid processing – including arginine and proline metabolism and alanine/aspartate/glutamate metabolism – which are critical for neurotransmitter synthesis (44). Abnormalities in aromatic amino acid and glycolytic pathways may also account for auditory hallucinations, a core domain of SCZ (45). A compromission of brain protection against oxidative stress is indicated by disrupted glutathione metabolism, with compromised antioxidant defenses and increased vulnerability to oxidative stress (44). Energy-related processes show widespread alterations, including galactose metabolism, glyoxylate/dicarboxylate metabolism, starch/sucrose metabolism, and pantothenate/CoA biosynthesis, suggesting impairment in the brain’s energy production and utilization. Lipid signaling processes – particularly unsaturated fatty acid biosynthesis – are also disrupted, potentially affecting neuronal membrane integrity and, subsequently, downstream signal transduction. Cognitive dysfunction, another core domain of SCZ, has been linked to alterations in neuronal energy metabolism, mitochondrial function, and neurotransmitter cycling (46, 47). Effective antipsychotic treatment seems to modulate many compounds, including several fatty acids, tyrosine, tryptophan, uric acid, lactate, aspartate, glycine, and myo-inositol, indicating partial normalization of brain metabolism as a marker of therapeutic response (48, 49).

As regards bipolar disorder (BD), metabolomic studies have shown widespread alterations in amino acid and lipid metabolisms, energy production, and neurotransmission that underpins both shared and phase-specific pathophysiological mechanisms. Several perturbations in branched-chain (valine, leucine, isoleucine) and aromatic (phenylalanine, tyrosine) amino acid metabolism (50) have been observed. The glycine/serine/threonine pathway seems consistently dysregulated across studies (51), influencing one-carbon metabolism, redox homeostasis, energy production, and cellular signaling. Metabolomics analyses have also confirmed altered tryptophan catabolism in BD (52), with decreased kynurenic acid and increased quinolinic acid in plasma (53). Lipidomics investigations have uncovered decreased plasmalogens and acyl-carnitines alongside elevated triacylglycerols, reflecting mitochondrial dysfunction and altered fatty acid β-oxidation (54). Pathway enrichment analyses have consistently observed disruptions in the tricarboxylic acid (TCA) cycle and glycolysis/gluconeogenesis have been constantly highlighted by blood studies (55) and corroborated by CSF investigations (56), comprehensively pointing towards impaired bioenergetic homeostasis in BD. All these core metabolism dysfunctions are accompanied by phase-specific metabolic signatures that, although stemming from a cross-sectional evidence base, may help to subtype episodes: bipolar depressive episodes show impaired glucose utilization and increased reliance on alternative fuels (β-glucose, glycerol, lactate, acetoacetate, lipids), manic/hypomanic episodes exhibit gut-microbiome and creatine-related shifts, and mixed episodes feature glycine/serine/threonine pathway disruptions (57). Despite the small number of available studies, evidence suggests that metabolomic profiles in subjects with BD seem influenced by psychotropic medications: for instance, lithium treatment resulted in elevated serum levels of L-lactic acid and 3-hydroxymethylglutaric acid compared to second-generation antipsychotic-treated subjects, while reducing linoleic acid and N-acetylglutamic acid concentrations (58).

Metabolomics studies of major depressive disorder (MDD) have consistently revealed perturbations across amino acid, energy-metabolism, lipid, and neurotransmitter pathways. Two-thirds of blood metabolomics studies in MDD converge on decreased levels of many amino acids: tyrosine metabolism and valine, leucine, and isoleucine biosynthesis are constantly among the most perturbed mechanisms, underscoring aromatic and branched-chain amino-acid disruptions (59). The glycine/serine/threonine pathway is also disrupted, implicating one-carbon and redox homeostasis alterations (60). Notable deficits in core bioenergetic processes are observed as well (59): significant perturbations in the TCA cycle (with decreased intermediates pyruvate and fumarate), glyoxylate/dicarboxylate metabolism, and butanoate metabolism indicate widespread energy metabolism dysfunction (61, 62). Consistent changes in phosphatidylethanolamines, lysophosphatidylcholines, ceramides, sphingomyelins, long-chain unsaturated lipids, and pantothenate/CoA biosynthesis also point to membrane alterations, abnormal fatty-acid oxidation, and mitochondrial disfunction (63, 64). Imbalances in excitatory-inhibitory mechanisms, with elevated glutamate and γ-aminobutyric acid (GABA), and reduced glutamine (59), could implicate increased neuronal glutaminase activity or impaired astrocytic uptake, creating a paradox of neurotransmitter excess despite precursor depletion (65). Altered kynurenine pathway (66), with decreased tryptophan, kynurenine and kynurenic acid, along with quinolinic acid, further implicate neuroinflammation and glutamatergic excitotoxicity in MDD, correlating with symptom severity and cognitive deficits (67). Both first-episode drug-naïve MDD (68) and treatment-resistant MDD (69) show peculiar metabolomics features. Antidepressant treatment seems to influence the metabolome, reversing the reduction of brain neurotransmitters caused by depression, modulating inflammatory activation and tryptophan catabolism, and alleviating abnormalities of amino acid, energy, and lipid metabolisms (70), with different metabolites correlating with improved symptoms (71). Notably, ketamine and esketamine modulate several metabolic pathways, including the TCA cycle, glycolysis, the pentose phosphate pathway, lipid metabolism, amino acid metabolism, the kynurenine pathway, and the urea cycle (72).

Metabolomics studies in anxiety disorders underscore perturbations in lipid homeostasis, amino-acid turnover, and TCA-related bioenergetics as promising biomarker candidates. Subjects with anxiety exhibit elevated phosphatidylcholines and ceramides, indicating possible disruption of neuronal membrane integrity, neurotransmitter synthesis, and signal transduction (73). As well, phenylalanine, tyrosine and tryptophan biosynthesis may be related to anxiety disorders (74). Urinary metabolomic profiling also revealed significant alterations in amino acid turnover, tryptophan catabolism and TCA cycle intermediates (73). However, given the high comorbidity between anxiety and depression, metabolomic signatures may partly reflect depressive symptomatology rather than anxiety-specific biology (75). Human studies of the effect of anxiolytic treatments on metabolomics features are lacking.

Metabolic signatures of neuroinflammation, oxidative stress, and bioenergetic deficits have been found in post-traumatic stress disorder (PTSD) among World Trade Center survivors (76) and veterans (77), with evidence of altered glycolytic, TCA, sphingolipid, glutathione, branched-chain amino-acid, and fatty-acid metabolisms. Divergent pathway activation patterns between recent versus chronic PTSD have been found (78). Specific blood metabolites (such as theophylline) have been linked with the risk of developing PTSD (79).

Metabolomic profiling of drug-naïve adults with obsessive-compulsive disorder (OCD) has revealed significant alterations in unsaturated fatty acid, tryptophan metabolism, and glutamate and GABA precursors (80, 81). Notably, plasma docosapentaenoic acid and 5-hydroxytryptophan levels may represent predictors of response to treatment with sertraline (81). These findings are partially substantiated by preclinical evidence (82).

A synthesis of main findings from metabolomics studies in mental disorders is reported in **Table 3**.

**Table 3:** Synthesis of findings from metabolomics studies across main mental disorders.

Disorder	Main disrupted pathways/metabolites	Medication effects
Schizophrenia	• Arginine and proline metabolism • Alanine/aspartate/glutamate metabolism • Galactose metabolism • Glyoxylate/dicarboxylate metabolism • Starch/sucrose metabolism • Pantothenate/CoA biosynthesis • Unsaturated fatty acid biosynthesis • Glutathione metabolism	Antipsychotics: modulate fatty acids, tyrosine, tryptophan, uric acid, lactate, aspartate, glycine, myo-inositol.
Bipolar disorder	• Branched-chain amino acid metabolism • Aromatic amino acid metabolism • Glycine/serine/threonine pathway • β-oxidation • Kynurenine pathway	Lithium: increases of serum levels of L-lactic acid and 3-hydroxymethylglutaric acid and decreases linoleic acid and N-acetylglutamic acid concentrations compared to second-generation antipsychotics.
Major depressive disorder	• Branched-chain amino acid metabolism • Aromatic amino acid metabolism • Glycine/serine/threonine pathway • TCA cycle • Glyoxylate/dicarboxylate metabolism • Butanoate metabolism • Membrane lipid homeostasis • Glutamate/GABA-glutamine cycle • Kynurenine pathway	Serotonergic antidepressants: reverse brain neurotransmitter reduction; modulate inflammation and tryptophan catabolism; reduce abnormalities of amino acid, energy, and lipid metabolisms. Ketamine and esketamine: modulate TCA cycle, glycolysis, the pentose phosphate pathway, lipid metabolism, amino acid metabolism, kynurenine pathway, and urea cycle.
Anxiety disorders	• Amino acid turnover • TCA cycle • Phosphatidylcholines and ceramides metabolism • Kynurenine pathway	Limited data on treatment effects.
Post-traumatic stress disorder	• Branched-chain amino-acid metabolism • Glycolysis • TCA cycle • Sphingolipid metabolism • Fatty-acid metabolism • Glutathione metabolism	Limited data on treatment effects.
Obsessive-compulsive disorder	• Unsaturated fatty acid metabolism • Glutamate and GABA synthesis • Tryptophan metabolism	Sertraline: docosapentaenoic acid and 5-hydroxytryptophan levels may represent predictors of response.

CoA, coenzyme A; GABA, γ-aminobutyric acid; TCA, tricarboxylic acid.

## 4 Interpretation, strengths, and limitations of metabolomics evidence in psychiatry

A convergent involvement of several pathways across different mental disorders emerges from this body of evidence. Perturbations in branched−chain and aromatic amino acids, together with changes in glutamate, GABA, glycine/serine/threonine and related intermediates, indicate altered neurotransmission (83, 84) across psychosis, mood, anxiety, and compulsivity (85, 86). In parallel, dysregulation of one−carbon and redox−related amino acid pathways may account for gene−expression regulation and vulnerability to oxidative damage observed in psychiatric disorders (87, 88). Consistent abnormalities in glycolysis, TCA cycle intermediates, related anaplerotic routes and markers of high−energy phosphates point to bioenergetics abnormalities (89, 90) that possibly contribute to cognitive symptoms, anergia, and diminished resilience. Alterations in acylcarnitines and other fatty acid-related metabolites additionally suggest that the capacity to flexibly switch between energy sources may be impaired in at least a subset of patients (91). Lipidomic findings are in line with disruptions of membrane homeostasis and lipid−mediated signaling (92). Finally, consistent involvement of tryptophan catabolism supports increased neurotoxicity (93).

Overall, metabolomics studies suggest that such biochemical alterations – largely shared across disorders – likely reflect both a genuine transdiagnostic pathophysiology, with convergent metabolic perturbations aligning with dimensional constructs that transcend diagnostic boundaries, and nosological shortcomings, with high diagnostic comorbidity and symptom overlap not cleanly separating distinct pathophysiologies. Also, most studies focus on SCZ, BD, and MDD, and findings are relatively well-replicated in multiple independent cohorts, providing an evidence base that supports biological validity and potential clinical utility. On the other hand, evidence for anxiety disorders, OCD, and PTSD remains preliminary, typically based on no more than three primary studies with limited cross-study replication. Sample sizes are small and often insufficient: while pilot discovery screens may require only 20–30 participants per group, validation studies demand substantially larger cohorts (~300 subjects for 0.95 power in multivariate settings), yet most investigations fall short of these thresholds (94, 95). First-episode, medication-naïve samples and stratification by drug class should be prioritized. The predominance of cross‐sectional designs further limits the differentiation of trait biomarkers from state‐dependent or treatment‐induced metabolic changes, not allowing a thorough characterization of temporal dynamics.

Lack of independent replication and external validation undermines the generalizability of reported findings. The reliance on peripheral biofluids introduces uncertainty about the correspondence between systemic and central biochemistry. Residual confounding by lifestyle factors, adiposity, smoking and medication can all generate apparent similarity in metabolite profiles. In particular, treatment-response studies are scarce and largely observational (96), limiting causal inference about whether metabolic shifts reflect therapeutic mechanisms or drug confounding. Pre‐analytical variables (biofluid type, fasting status and sampling/storage/processing protocols) vary substantially (97), as do analytical platforms: NMR is limited by lower sensitivity and resolution (23), while MS can suffer from variability that affects reproducibility (31). High-dimensional metabolomics data, with the number of variables greatly exceeding the number of samples [the “curse of dimensionality” (98)], require complex pre-processing and pose the risk of model overfitting. This requires robust computational frameworks (99): advancing data analysis, for instance implementing advanced ML approaches, can enhance phenotype discrimination and produce more refined predictive models (100). Explainable AI models (101, 102) and open data sharing (103) can improve interpretability and accelerate validation efforts.

Therefore, while metabolomics offers unprecedented insights into the molecular underpinnings of mental disorders by providing objective, high-throughput profiling of small-molecule metabolites, laying the groundwork for precision psychiatry, this literature is best interpreted as promising but preliminary.

Although there is recurrent evidence for small, biologically anchored panels (e.g., those involving amino acids and kynurenine metabolites), only a few metabolomic signatures in psychiatry can currently be considered “near translational”, and none is yet ready for routine clinical use. Candidate metabolites/panels still face major barriers: untargeted workflows are costly and technically demanding, targeted assays are not yet standardized across laboratories, and no study has conclusively shown that adding metabolomics testing to existing clinical assessments improves outcomes in a cost-effective way. Combining complementary techniques can overcome the limits of single analytical platforms, yielding more comprehensive biomarker panels (26, 50). Moreover, multi-omics approaches, jointly analyzing metabolomics with genomics, transcriptomics, and proteomics, can generate robust composite signatures that surpass single-omics to yield deeper mechanistic insights (99, 104), and integrating metabolomics with genetics, neuroimaging and digital data can provide multimodal combinations that are more likely to yield clinically useful stratification and prediction than any single modality alone (105, 106).

In general, study designs should be tailored to clinical utility, focusing on prospective, outcome-oriented biomarker validation to ensure that findings are directly translatable into meaningful clinical interventions, for instance identifying metabolomics predictors of treatment response (107).

## 5 Conclusions

Metabolomics offers unprecedented insights into the molecular underpinnings of mental disorders by providing objective, high-throughput profiling of small-molecule metabolites, laying the groundwork for precision psychiatry. The high dimensionality of metabolomics data has the potential to be used to identify metabolic signatures that distinguish diagnostic groups and predict symptom trajectories, especially when considered not as isolated markers but within panels. In this direction, composite metabolite scores and pathway enrichment further help gain insights into metabolic abnormalities (44). Beyond diagnostics, metabolomics monitoring can offer a dynamic tool for individualized treatment optimization by tracking biochemical responses to pharmacotherapy (96). Comprehensively, metabolomics promises to deliver tailored diagnostic panels, adaptive monitoring strategies, and mechanism-driven therapies that could move psychiatry beyond trial-and-error prescribing towards truly personalized mental health care.

Metabolomics has matured from small, proof-of-principle studies to large multi-platform investigations that consistently implicate several biochemical domains across psychiatric disorders. To date, studies have identified alterations in amino acid, lipid, energy, and inflammatory pathways in severe mental disorders. Yet, despite the encouraging evidence, the field faces substantial hurdles, with methodological challenges currently limiting harmonization and clinical translation of these findings. When these components are in place, metabolomics can become a key pillar of biologically informed, personalized psychiatric care, improving diagnosis, subtype stratification, treatment, and clinical monitoring.
